# Evaluating Partnerships to Prevent and Manage Chronic Disease

**Published:** 2009-03-15

**Authors:** Frances Dunn Butterfoss

**Affiliations:** Coalitions Work

## Abstract

To be effective and sustain themselves over time, public-private partnerships must make evaluation a priority. Specifically, partnerships should evaluate 1) their infrastructure, function, and processes; 2) programs designed to achieve their mission, goals, and objectives; and 3) changes in health and social status, organizations, systems, and the broader community. This article describes how to 1) develop a comprehensive evaluation strategy based on partnership theory; 2) select short-term, intermediate, and long-term indicators to measure outcomes; 3) choose appropriate methods and tools; and 4) use evaluation results to provide accountability to stakeholders and improve partnership function and program implementation.

## Introduction

Public-private partnerships fundamentally bring together the expertise of the private and public sectors and allow each to do what it does best, so that products and services can be delivered efficiently and effectively. These partnerships also can help overcome organizational boundaries and allow parties to work together on a shared goal. For example, even though collaborating with private-sector businesses may cause tension among nonprofit public-sector partners, the businesses may bring new skills and funding to the partnership and enhance the partnership's scope of influence. An illustration: 3 partnerships were sponsored by a national association of health plans to improve the quality of diabetes care in New Mexico, Missouri, and New York. The main evaluation finding was that the competing health plans and local organizations established and sustained viable partnerships around a shared goal, despite significant challenges (1). The process of evaluation discovered the effects of these vital partnerships by answering the following questions: 1) What attributes and events resulted in competing organizations coming together? 2) What is necessary to sustain these partnerships? 3) What recommendations can help replicate this approach for other chronic conditions in other communities? and 4) To what extent do local market characteristics and structures set partnership direction and influence success?

Evaluation is a critical task for any partnership and determines whether the organization and its activities are sustained over time. Effective evaluation provides ongoing, systematic information that strengthens the partnership during implementation and provides outcome data to assess the extent of change among participants or within systems (2).

Both the for-profit and nonprofit sectors place a high value on evaluation and regard it as a necessity rather than an enhancement. However, their underlying value systems and motivating factors may differ. For the nonprofit sector, partnership evaluation fulfills underlying process goals, such as identifying new approaches, increasing community awareness and support, informing policy decisions, and contributing to the understanding of what works (3). The public health issues have developed over time, and the public health community's expectations are that desired evaluation outcomes may take years or even decades to accomplish. Conversely, the for-profit sector is driven by increasing its market share, improving its performance through continuous innovation, having good financial management and stability, increasing efficiency, increasing customer accountability and satisfaction, improving its access to government officials, and improving public relations. The time horizon for outcomes in the business sector is often a few months to 2 years. As the value systems of the for-profit and nonprofit sectors converge, evaluation also will become more of a shared value. Partnering certainly can enable this process.

This article describes how evaluation is viewed by nonprofit and for-profit sectors, levels of partnership evaluation, and a step-by-step model for evaluating partnerships. I conclude by presenting the challenges to evaluating partnerships and recommending solutions.

## Views on Participatory Approaches to Evaluating Partnerships

Many partnership evaluations are based on collaborative or participatory approaches according to who controls the process, who participates, and how much (4). These approaches are well suited to partnerships, although they have disadvantages (Appendix A) (5).

Participatory approaches to evaluation are generally comfortable and customary for the public sector. The private sector may not be as familiar with these types of evaluations nor as patient with the extra time and effort that it takes to be democratic and attentive to the needs of all partners, priority populations, and communities served. Discussing the extra benefits that result from such evaluations, such as better understanding and acceptance of findings that may improve performance, may enable for-profit partners to be more open to these approaches and learn by participating in the evaluation process (6).

When diverse partners work together, evaluation approaches and terms must be clarified. The private sector and the public sector may differ in their approaches to the evaluation or assessment process, the standards and methods they use to gather data, how they define terms, and the kinds of indicators they plan to measure.

For example, the VERB campaign used the best practices of private-sector marketing to children. The VERB brand created an emotional affinity between the product (physical activity) and the user (tween), and engaged tweens at key places and times when they might be both inactive and receptive to the brand (7). The public sector redefined its terms — education had to include *persuasion*, VERB was not a program but a *brand*, and pooling resources with the private sector allowed the public agency, the Centers for Disease Control and Prevention (CDC), to buy media time and the talents of marketing experts. The agency also adjusted its concepts of performance measures and outcomes to meet both private investors' needs and public health's goals.

Coming to consensus on definition of terms, methods, and measures is a crucial step in building trust when beginning a partnership evaluation. Although public health professionals may feel comfortable using the terms *process*, *impact*, and *outcome* *measures*, using the terms *short-term*, *intermediate*, and *long-term indicators* is more descriptive and avoids confusion when working with partners from diverse professional backgrounds. Different evaluation terms that the for-profit and nonprofit sectors may use in a public-private partnership are listed in Table 1.

## Levels of Partnership Evaluation

In public-private partnerships, evaluation may measure 1) processes that sustain and renew partnership infrastructure and function; 2) programs intended to accomplish targeted activities or those that work directly toward the partnership's goals; and 3) changes in health status or the community. Appendix B details these 3 levels and sample measures for each. The aim of every partnership should be to evaluate something in each level. Conducting a member survey to assess satisfaction with how the organization functions (level 1), evaluating 1 program or activity that the partnership conducts (level 2), and collecting extant data on key health indicators (level 3) are reasonable expectations for an annual evaluation plan.

## Steps in Partnership Evaluation

Many practical frameworks and models exist that can help partnerships develop evaluation plans, but the focus here is on the Framework for Program Evaluation in Public Health (8,9). The framework guides its users in selecting evaluation strategies that are useful, feasible, ethical, and accurate — its 6 steps help increase understanding of a partnership's context as well as its outcomes. The steps have been refined and described below to apply to partnership evaluation.

### Step 1: Engage the stakeholders

Engaging stakeholders means fostering participation and power sharing among people invested in the evaluation and its findings. Stakeholders include 1) those involved in program operations (eg, partners, public relations professionals, lawyers, sponsors, funders, collaborators, administrators, managers, business owners, staff), 2) those served or affected by the program (eg, clients, customers, families, neighborhood organizations, academic institutions, elected officials, advocacy groups, professional associations, opponents), and 3) primary users of the evaluation. Stakeholders must understand the organizational structure, history, and goals of the partnership and how politics affect program implementation and impact. This understanding can be attained by creating an environment where stakeholders discuss their values, philosophies and assumptions, and capabilities. Stakeholders may 1) provide resources for evaluation such as staff and in-kind supplies, 2) clarify partnership goals and objectives, 3) identify and prioritize evaluation questions, 4) develop and pilot evaluation methods and tools, 5) collect data, and 6) interpret and report results (10).

Stakeholders' needs, concerns, and demands for specific outcomes differ widely, even though they may agree with the partnership's goals and objectives. To motivate the stakeholders to participate in the partnership and its evaluation, data could be gathered from them about what they need the evaluation to measure. A sample set of criteria is included in Table 2 (11).

### Step 2: Describe the partnership

This description should focus on the purpose, goals, objectives, resources, current and planned activities, expected outcomes, stage of development, and environmental context of the partnership. In a public-private partnership, objectives are based on compromise among partners with different political, social, and economic aims. Divergent interests concerning actions and expected impacts must be taken into account (11). Developing a logic model is 1 way to help stakeholders clarify the partnership's rationale, strategies, and conditions. It serves as a road map of the program, prioritizes the sequence of activities, summarizes expected change by linking processes to eventual outcomes, shows how partnership programs are linked to other ongoing efforts, and displays the infrastructure needed to support the partnership (9). An effective logic model will be refined and changed many times, as the partners learn about how and why the partnership works. A sample logic model for a state partnership is in CDC's National Heart Disease and Stroke Prevention Program's Evaluation Guide (10).

Partnerships develop in stages: 1) formation — initial building of the organization, 2) implementation — strategic planning and conducting of activities to address goals, 3) maintenance — sustaining activities until goals are accomplished, and 4) institutionalization — collaborative attainment of goals in permanent structures within the community (12,13). The current stage of the partnership should be assessed to determine the proper focus for evaluation (8). For example, evaluation activities should focus on identifying and recruiting partners if the partnership is in the formation stage; communication and decision making in the maintenance stage; and community changes in the institutionalization stage. A comprehensive evaluation of a mature partnership includes measures at each stage.

### Step 3: Focus the evaluation design

The evaluation should focus on issues of greatest concern to stakeholders, while efficiently using time and resources. A written plan that summarizes evaluation goals and procedures and outlines the stakeholders' roles and responsibilities is essential. The plan should include evaluation questions and practical methods for sampling, data collection, data analysis, and interpretation. Stakeholders can help prioritize the questions to determine which are critical, are likely to improve the partnership, and can be answered with available resources. Questions may include the following:

- What should the partnership accomplish and how will it be demonstrated?
- What activities will the partnership undertake to accomplish its goals?
- What factors might help or hinder the accomplishment of its goals?
- Who are the partners (number, diversity, and participation levels)?
- How do partners work together?
- What partnership outcomes should be measured?

The evaluation design is linked to the priority questions, and the choice of design has implications for what data will be collected and how. A pretest-posttest design uses a comparison group, measures the partnership on given parameters before and after it implements planned improvement strategies, or both. A case study design is used to study the partnership's context, history, structure, and function. Case studies usually rely on multiple sources of information such as observations, interviews, audiovisual material, documents, and reports. Appendix C provides a sample evaluation plan for partnerships (14).

### Step 4: Gather credible evidence

After deciding on the evaluation questions and design, the partnership must decide what data it needs to answer the questions, where and how the data can be obtained, and how the data should be analyzed and used. Adequate data may be available and easily accessed, or new data may have to be collected. Evaluation data should provide a well-rounded picture of the partnership and its programs so stakeholders can perceive the results as believable and relevant. Integrating qualitative and quantitative data increases the likelihood that data will be balanced and accepted by all stakeholders (15).

For each evaluation question, at least 1 indicator or data point must be defined and tracked. Examples of indicators for partnerships might include measures of 1) partnership effectiveness (eg, participation in meetings and activities, usefulness of partnership structures), 2) partnership activities (eg, participation rate, completion of state plan objectives), and 3) partnership effects (eg, number of policies or practices that were amended or adopted, health status changes). Practitioners and researchers have summarized measures that document changes in partnership knowledge, attitudes, practices, community environment, policies, and health status (2,16,17).

For each evaluation question and indicator, sources of data must be identified. Data from documents, key informant interviews, meeting observations, member surveys, and focus groups provide different perspectives of the partnership and enhance the comprehensiveness and credibility of the evaluation. Census data (including economic data and demographics), health survey data (eg, Behavioral Risk Factor Surveillance System survey results), or behavioral outcome data (eg, emergency medical transports, hospital admissions) represent likely data sets that partnerships may use to assess health and quality of life status. A rule of thumb is to collect only data that will be used and to use all data collected.

In deciding what instruments to use, partnerships may develop their own questionnaires or interview frameworks, use validated and reliable tools, or modify an existing tool to fit their priority population(s), community culture, and issues. *Coalitions and Partnerships in Community Health* (2) lists tools and resources for these evaluations. Stakeholders should develop clear procedures for gathering, analyzing, and interpreting data, and training staff, partnership members, and volunteers to collect quality data.

### Step 5: Justify conclusions

After designing an evaluation, data must be collected, described, analyzed, and synthesized to summarize the findings, then interpreted to decide what it means in the context of the partnership. Investing enough time and resources in analysis and interpretation is critical because this is when decisions are made and actions are taken. Once data are collected, they are returned to stakeholders for reflection and verification. Stakeholders should look beyond the raw data to ask what the results mean, what led to the findings, and whether they are significant. Each partner has different criteria for judging success and weighs them differently; using multirater analysis may help (11). Conclusions are justified and will be used with confidence when they are supported by data, consistent with the agreed-on values of the stakeholders, and linked to recommended actions.

### Step 6: Ensure use and share lessons learned

The partnership should provide continuous feedback to stakeholders regarding interim findings to ensure that evaluation conclusions lead to appropriate decisions or actions. Stakeholders are more likely to use evaluation results if they feel they own the evaluation process and if they function cohesively as a team. During each planning and implementation step, stakeholders should discuss the best ways to communicate evaluation findings and use them. Frequent communication will increase the commitment to act on the results and refine the evaluation design, questions, methods, and interpretations. Having a positive experience with evaluation changes participants' attitudes; they begin to base decisions on judgments instead of assumptions (6).

## The Challenges of Evaluating Partnerships

Researchers agree that partnerships are difficult to evaluate. Measuring system-level changes is more difficult than evaluating program outcomes because multiple levels and community readiness must be considered (18). Other practical and methodologic issues include the following:

- The partnership's planning process does not include evaluation. Resources are often inadequate or are more likely to be spent on interventions. Because evaluation is costly in time and resources, the partnership is not always committed to do it. If evaluation is supported, staff are motivated to make partnership programs look effective to maintain funding or jobs. Evaluation may not be based on a solid logic model or theory, and partnerships may fail to find the right evaluators or tools for evaluating partnership processes and outcomes (19).
- Each partnership is unique. Partnerships are embedded within communities and responsive to their cultural contexts. For this reason, they tend to be unique, difficult to replicate, and unrepresentative of other partnerships, even those that address similar issues.
- The design and methods of the evaluation can make generalization difficult. Establishing and measuring outcomes, controlling extraneous variables that interact with outcomes, accounting for secular trends over the partnership's development, and addressing the political realities to satisfy funders make it challenging to detect systems-level change (20). Extraneous variables (eg, new government programs, changes in funding streams) are difficult to control and may interact with each other or influence outcomes by changing how programs are implemented. Partnerships may not identify outcome measures or link them to appropriate intermediate outcomes. Even when comparable long-term outcomes are measured across sites, baseline data may not be available. Finally, distinguishing between cause and effect or the percentage of the outcome that can be attributed to each partnership activity is difficult (20-22).

## Recommendations to Improve Partnership Evaluation

Partnerships may be the best vehicles available to address the chronic diseases of our time, so evaluation methods continuously need to be refined. The following are proposed solutions to partnership evaluation issues (2,3,20,23-27):

- Use innovative, qualitative evaluation methods. Rely on qualitative methods that represent the community and try to figure out how partnerships make a difference. Innovative methods need to be developed to address the dynamic nature of partnerships (25).
- Focus on evaluating practice-proven strategies and measurable outcomes. Partnerships are best suited to assessment and priority setting rather than implementing projects (4). Evaluators must concern themselves with short-term, immediate, and long-term effects of the partnership. In addition to health and social outcomes, evaluation should focus on how partnerships build capacity by improving outcomes related to participation, leadership, networks, skills, resources, and sense of community (28). Similarly, evaluators may determine whether a partnership is on track to become empowered and sustained by noting outcomes such as community infrastructure improvements, economic enhancements, educational opportunities, and policy changes (29).
- Provide needed training and technical assistance. Appropriate training, technical assistance, and resources for conducting effective evaluations should be available to partnerships, so they learn how to translate evaluation results into actionable tasks.
- Help partnerships "begin where they are." Most partnerships view evaluation as a formidable task and choose not to evaluate. They are overwhelmed by technical tasks, time and financial costs, and concerns that they might not "measure up." Partnerships should be encouraged to start small and evaluate something. They might choose to evaluate 1 aspect of the partnership from each of 3 levels (short-term, intermediate, and long-term) as a starting point. Existing data can be evaluated with little or no cost. As examples, partner diversity can be determined by assessing the roster; attendance patterns can be derived from the meeting minutes. As confidence and skills grow, partners may be encouraged to engage in new and more complex evaluation tasks.

## Conclusion

Public-private partnerships can be powerful agents for preventing and managing chronic disease. However, such partnerships become more complex as the public sector works more closely with private-sector partners. The following issues must be considered in developing evaluations that lead to improvements in partnerships and their programs and services:

- What common evaluation criteria can be agreed on (eg, frequency of evaluation)?
- How can the environment for public-private partnerships be assessed?
- How can partnerships obtain adequate resources to conduct effective evaluations?
- What are the roles of various stakeholders in the evaluation? How can technical and evaluation capacity be fostered?
- How can evaluation be built into the framework of the partnership?
- Can existing tools and methods be adapted to meet public and private partners' needs?

How well these issues are addressed will help determine the effectiveness of partnership evaluations. Evaluations that meet stakeholder needs and focus on mutually acceptable and measurable systems-level outcomes will make partnership support and sustainability more likely in the end.

## Figures and Tables

**Table 1:** Evaluation Terms Commonly Used by Nonprofit (Public Sector) and For-Profit (Private Sector) Partners

**Nonprofit (Public Sector)**	**For-Profit (Private Sector)**
Evaluation	Assessment or monitoring
Program effectiveness	Efficiency or cost-effectiveness
Program or intervention	Product
Quality assurance	Quality improvement
Outcomes	Results or benchmarks
Process measures	Short-term indicators or benchmarks
Impact measures	Intermediate indicators or benchmarks
Outcome measures	Long-term indicators or bottom line
Priority populations	Targets or market segments

**Table 2:** Partnership Sectors and Relevant Evaluation Parameters^a^

**Partner Sector**	**Evaluation Criteria**
Economic/business partners	Job creation Employment and volunteer opportunities Personal income level
Human services partners	Access to essential services (eg, housing, sanitation, clean water, adequate nutrition)
Health partners	Population health status (eg, morbidity and mortality statistics) Health care access and treatment
Education partners	School enrollment School dropout rates Literacy rates
Human rights partners	Negative freedoms from forced labor; judicial killings; unlawful detention; or torture, coercion, and corporal punishment Positive freedoms to associate and assemble peacefully, organize political opposition and trade unions, and speak freely and participate in public debates
Government and political partners	Administrative capacity or organizational development and strengthening to improve service delivery Capacity to plan, implement projects, and act as pressure group to gain influence Financial and human resources

a Source: Toulemond et al (11).

## Appendices

### Appendix A. Advantages and Disadvantages of Participatory Evaluation of Public-Private Partnerships

Advantages

- Engages and empowers multiple stakeholders.
- Improves program implementation and outcomes.
- Uses systematic, multidisciplinary approaches to problem solving.
- Is based on local community circumstances and issues.
- Is flexible; adapts to evolving needs of organization and its projects.
- Recognizes power of participation and works to enhance or sustain it through group dialogue, training, and action.
- Provides broader feedback of higher quality.
- Provides better understanding and acceptance of findings.
- Provides practical recommendations and reports that increase likelihood that evaluation results will be used.

Disadvantages

- Participants' motivation, commitment, and skills vary.
- Extra resources are needed to build relationships and train partners.
- Highly technical reports are not usually produced.
- Extra time is needed to fully involve members and obtain needed feedback.
- Some rigor may be lost initially.

### Appendix B. Partnership Evaluation Levels and Measures

**Level 1 — Partnership infrastructure, function, and processes**

In the early stages, partnerships create mission statements, set up work groups, conduct assessments, and develop action plans. To sustain momentum, a partnership has to recruit and orient new members, train leaders, prepare members to assume leadership when turnover occurs, address and resolve conflict, engage in public relations, raise funds, and celebrate its accomplishments. Process evaluation will document what was done, how people were recruited and engaged in partnership efforts, and whether the partnership is functioning optimally or as originally intended. This type of evaluation essentially assesses the short-term outcomes of the partnership's development as an organization. Collecting and analyzing annual reports, attendance records, contribution records, meeting minutes, activity logs, and surveys that measure members' levels of satisfaction, commitment, and participation are methods and measures that may be used to document partnership structure and function.

Level 1 measures are short-term outcome measures used to document changes in the number and type of partners, the perceptions or skills of staff and members, or the mission or direction of the partnership.

- ***Member representation**.* Representation of organizational partners by community sector, diversity of racial and ethnic groups, and members' perceptions of representativeness.
- ***Member skills and experience.*** Number of years worked on issue, skills and expertise related to issue, collaboration and partnership management skills, and other strengths.
- ***Recruitment.*** Number of community sectors represented, average length of membership, success in recruiting new members, and steps taken to ensure representativeness.
- ***Participation.*** Length of service; level of participation over time; average meeting attendance; number of meetings and activities attended; dropout and retention rates; number of hours spent per month on partnership work; service on committees or in leadership roles; voluntary, paid, or consultant roles; types of activities engaged in; extent of personal and organizational contributions; and effective use of member abilities.
- ***Role clarity.*** Knowledge about partnership mission, structure, and operations, and whether role perception of members matches that of staff about partnership's involvement in developing action plans, budgets, and plans and objectives.
- ***Costs and benefits of participation.*** Costs include personal or partnership/group difficulties such as personal, social, material, and purposive costs. Benefits include personal, social, purposive, and material benefits, and extent to which participation changed members' knowledge, beliefs, and skills. Overall assessment of benefits versus costs of participation also can be made.
- ***Satisfaction.*** Global satisfaction with work of partnership and with personal involvement, team's work and plan, specific aspects of group function and achievement, and progress and strength of organization.
- ***Commitment.*** Strength of member commitment to partnership, sense of pride, endorsement of mission and efforts, and caring about its future.
- ***Collaboration.*** Increased cooperation, joint planning of activities, networking, and information exchange. Determining factors include sharing a vision, political climate, history, understanding of goals and objectives, roles and responsibilities, decision-making processes, connectedness, conflict management, leadership, plans, policies, relationships, trust, community development, communication, resources, and evaluation.
- ***Sense of ownership.*** Staff and partners' commitment, sense of pride, and concern for partnership's future; perceived influence on organizational processes, goals, and structure.
- ***Sense of community.*** Feelings of connection, support, and collective problem solving; perceived severity of community problems.
- ***Expectations.*** Expectation that partnership will have effect on health issue, partners will engage in activities, planned activities will be fully implemented, and group will accomplish planned outcomes.
- ***Perceived effectiveness.*** Perception that partnership is effective in its activities, fund raising, coordination, training, goal setting, communication, public relations, and evaluation.
- ***Leadership.*** Leader support style (egalitarian, empowering, encouraging), decision-making style and control (democratic or authoritarian), competence, effectiveness in articulating vision, decision making, incentive management, conflict management; defining roles, facilitating meetings, nurturing collaborative group commitment and achievement, providing guidance, support, and feedback.
- ***Staff performance.*** Staff time devoted to partnership; staff expertise, priorities, interest, availability and turnover; transfer of knowledge and skills from staff to members; staff relationship with members; efficiency in managing partnership process and operating procedures; staff ability to guide and support partnership; staff shares responsibility with members; and staff costs and benefits of maintaining partnership.
- ***Formal organizational structure and planning products.*** Level of formality and complexity of partnership's structure and operation, as measured by assessing presence and quality of bylaws, agendas, minutes, and action plans; functioning steering committee and work groups, planning mechanisms, memoranda of agreement, and procedures for leadership stability and renewal; member orientation and training, communication, decision making, and conflict resolution.
- ***Task focus and meeting effectiveness.*** Task focus of meetings, order and organization of group, meeting effectiveness and efficiency, formal structures and accomplishment of tasks by stage of development.
- ***Organizational climate.*** Involvement, inclusion and task focus, organizational barriers, satisfaction level and commitment, cohesion, leader support and control, expression, independence, task orientation, self-discovery, anger and aggression, order and organization, and innovation within the partnership.
- ***Group relationships.*** Partnership relations or nature, quality and frequency of interactions; trust, conflict management, team work, use of talents, and recognition; and comfort and satisfaction of being heard and valued.
- ***Communication.*** Quality, frequency, and productivity of member-staff and member-member communications, and use of various communication methods.
- ***Conflict.*** Amount of tension in partnership caused by opinion differences, personality clashes, hidden agendas, and power struggles.
- ***Decision making.*** Extent of influence that individuals, group, staff, and leaders have in determining partnership's policies and actions, and member inclusion and involvement in group processes.
- ***Resources.*** Personnel, sponsorships, contracts, grants, funds, and in-kind donations mobilized by partnership and whether they are sufficient and effectively used.
- ***Plan quality.*** Evaluation of partnership's action or state plan and planning process — includes scope, comprehensiveness, clarity, effectiveness, and quality of plans. Specific measures include clear and achievable mission, goals, objectives and tasks, defined responsibilities, and use of community resources.
- ***General functioning.*** Partnership's capacity for group functioning and effective action related to community ownership, organizational effectiveness, group accomplishments, relationships between partners, other organizations, and community. Includes activities and factors involved in recruitment, goals, leadership, responsibilities, decision making, building trust, fund raising, making linkages and referrals, securing resources, strengthening policy or regulations, managing negotiations, cultural competence, planning, recruitment, training, evaluating, and building capacity.

**Level 2 — Partnership programs and interventions**

To achieve program outcomes, partnership activities (eg, training, advocacy, education programs) must be fully implemented and reach priority populations. For example, a partnership to prevent asthma might develop curricula, public awareness campaigns, or advocate changes in clean indoor air policies. Successful implementation depends on available resources, a time-phased action plan, and a supportive environment. This level of evaluation is conducted not only to prove that programs work but also to improve them. Adaptations of interventions that have been previously evaluated or accepted as promising practices increase the likelihood that interventions will result in systems-level change, and ultimately, desired health and social outcomes. Intermediate outcomes that are associated with changes in the priority population, the partnership, or the state's capacity to achieve long-term outcomes should be measured. For example, observations of clinic functioning, home visit logs, media reach reports, and event attendance forms provide short-term evidence of whether programs are implemented effectively and with fidelity. Patient record reviews help determine whether medical personnel adopt practice guidelines, and legislative records provide evidence of when and how proposed policies were introduced or passed.

Level 2 measures are short-term and intermediate outcomes that focus on activities and programs that the partnership accomplishes; the people, organizations, and groups it serves and affects; and the scope of the efforts it initiates. These measures include accomplishment of program outcomes such as changes in knowledge, attitudes, and behaviors.

- ***Implementation.*** Overall assessment of extent of implementation of action plan, type and number of completed activities, resources generated, and capitalization of opportunities outside of plan.
- ***Media coverage.*** Coverage of partnership events or issues by radio, television, and print media (number of radio spots, number of news outlets featuring the advertisements, amount of time recorded, or inches of print).
- ***New or modified services or programs.*** Classes, programs, workshops, publications, services, or communications provided by partnership and members.
- ***Community actions taken.*** Actions taken to encourage community change, such as promoting multiple administrations of vaccines or creating a safety checklist to monitor condition of playground equipment.
- ***New or modified policies.*** Policies promoted by partnership and its members, such as restricting sales of cigarettes to minors or enacting smoke-free restaurant ordinance.
- ***New or modified practices.*** Practices promoted by partnership and its members, such as following standard pathway to improve emergency department treatment of childhood asthma.

**Level 3 — Health and systems change outcomes**

For partners and funders, the bottom line is whether the partnership achieves its ultimate, long-term goals. Systems change does not happen quickly, and many outcomes are difficult to measure using traditional quantitative methods. Participatory and qualitative evaluation methods increase understanding about how and why community-based initiatives work. Epidemiologic data will indicate whether health status indicators have changed, but key informants can identify the partnership programs that are institutionalized within their organizations.

Level 3 measures focus on ultimate partnership outcomes beyond programmatic activities — long-term outcomes such as changes in health status, quality of care, effectiveness of community institutions, and overall changes in the community's capacity and competence to deal with emerging problems.

- ***Community capacity.*** Community's ability to plan, solve problems, implement programs, network, collaborate, conduct research, and evaluate its work.
- ***Organizational viability.*** Assessment of whether groups continue to meet and function, level of institutionalization of programs, organizational empowerment, and community competence.
- ***Health status indicators.*** Ultimate changes that relate to partnership's mission. A chronic disease prevention partnership would expect reduced rates of cardiovascular disease and cancer; a partnership focused on asthma management would expect reduced incidence of asthma exacerbations and hospitalizations and emergency department admissions due to asthma.

### Appendix C. Partnership Evaluation Questions and Methods

**Table:**

Questions (Evaluation Measure)	Type of Data Collection					Type of Design		

	Survey/ Scale	Structured Interview	Self-Report/ Log	Direct Obser-vation	Archival Records	Case Study	Pretest-Posttest Control Group	Time Series
**Planning and implementation issues (descriptive and process measures)**								
Who participates? (demographic data)		X	X			X		
Why do partners drop out? (partners' reasons for dropping out)		X	X			X		
Are different activities generated? (type and frequency of activities)				X	X	X		
**Assessing attainment of objectives (outcome measures)**								
How many participate? (no. of partners)			X	X	X	X		X
How many hours are partners involved? (no. of hours by activity)			X	X	X	X		X
How many people are trained? (no. of partners per workshop/retreat)			X	X	X	X		X
**Impact on participants**								
How do attitudes and behavior change by participating in program? (changes in attitude and behavior)	X	X	X	X	X		X	X
Does participation affect incidence, prevalence, or management of disease? (incidence/prevalence of asthma, diabetes, heart disease, and stroke)	X	X			X		X	
Are participants satisfied with experience? (satisfaction ratings)	X					X		
**Impact on community**								
What resulted from program? (changes in programs, policies, and practices of partner organizations)	X	X	X	X	X			X
Do partnership benefits outweigh costs? (cost-benefit data)		X	X		X	X		
Are community members satisfied with partnership and services they provide? (beneficiaries and community members/ satisfaction ratings)	X					X		

a Adapted from Francisco et al (15).
